# Masson’s Hemangioma of the Vastus Medialis: A Rare Case Involving the Musculoarticular Branch of the Descending Genicular Artery

**DOI:** 10.7759/cureus.86038

**Published:** 2025-06-15

**Authors:** Christos Lyrtzis, Alexandra Arhonidu, Nikolaos Anastasopoulos, Anastasia Fotiadou, George Paraskevas

**Affiliations:** 1 Department of Anatomy and Surgical Anatomy, Faculty of Health Sciences, Medical School, Aristotle University of Thessaloniki, Thessaloniki, GRC; 2 Department of Anatomy, Faculty of Health Science, Aristotle University of Thessaloniki, Thessaloniki, GRC; 3 Department of Pathology, Laboratory of Diagnostic Histopathology, Thessaloniki, GRC

**Keywords:** descending genicular artery, intramuscular mass, masson’s hemangioma, untraarticular mass, vastus medialis

## Abstract

Masson’s hemangioma, or intravascular papillary endothelial hyperplasia (IPEH), is a rare, benign vascular lesion that can mimic malignancies such as angiosarcoma. Although commonly found in the head, neck, and extremities, intramuscular involvement near joints is unusual. This report presents a rare case of IPEH originating within the vastus medialis muscle and involving the musculoarticular branch of the descending genicular artery in an adolescent male.

An 18-year-old male presented with right knee pain and a palpable mass without prior trauma or systemic disease. MRI revealed a vascular lesion within the vastus medialis in proximity to the knee joint. Surgical excision was performed, and histopathology confirmed the diagnosis of IPEH with no malignant features. Postoperatively, the patient regained full knee function without recurrence at 10-month follow-up.

Recognition of IPEH in atypical intramuscular and periarticular locations is essential to avoid misdiagnosis and overtreatment. Histopathological examination remains the cornerstone for distinguishing this benign entity from aggressive vascular malignancies.

## Introduction

Masson's hemangioma, also referred to as intravascular papillary endothelial hyperplasia (IPEH), is a rare, benign, and unusual non-neoplastic vascular lesion. It is also called Masson's tumor, because Masson in 1923, prescribed it [1]. It is a form of neoplasm, which is explained as a proliferation of endothelial cells into the vessel lumen, followed by obstruction and secondary degeneration, and necrosis. It is characterized histologically by papillary fronds lined by proliferating endothelium [1]. The lesion can occur anywhere in the body, but it most commonly affects the head, neck, and extremities [2]. There are different descriptions of its pathophysiology. It may present a proliferation of endothelial cells into the vessel lumen, followed by obstruction and secondary degeneration and necrosis. Alternatively, it may be a reactive process rather than a neoplasm, as the endothelial proliferation forms benign papillary structures and the cells show no atypia. Today, it is considered to be a reactive vascular proliferation following traumatic vascular stasis [1].

It is classified into three types. The first type and most common of IPEH arises de novo in distended blood vessels, typically veins, and is characterized by dilated vascular spaces. The second, mixed form of IPEH develops secondarily within pre-existing vascular lesions, such as hemangiomas, pyogenic granulomas, and lymphangiomas. Finally, the third, least common and undetermined form arises extravascularly within a posttraumatic hematoma and is often associated with trauma [3].

Diagnosing IPEH is challenging due to its microscopic similarities to angiosarcoma; therefore, biopsy and histopathological examination are performed post-surgery for accurate diagnosis [4]. Cytological examination may also be performed to assess the potential for malignancy. If bone or joint involvement is suspected, imaging techniques such as magnetic resonance imaging (MRI) and computed tomography (CT) are used to detect lesions. Immunohistochemistry is not recommended for diagnosis due to its inability to differentiate between IPEH and angiosarcoma [5].

We report a rare case of an 18-year-old Caucasian male who underwent wide resection of a soft-tissue tumor in the lower thigh, later identified histopathologically as a benign intravascular papillary endothelial hyperplasia involving the musculoarticular branch of the descending geniculate artery.

## Case presentation

**Figure 1:**
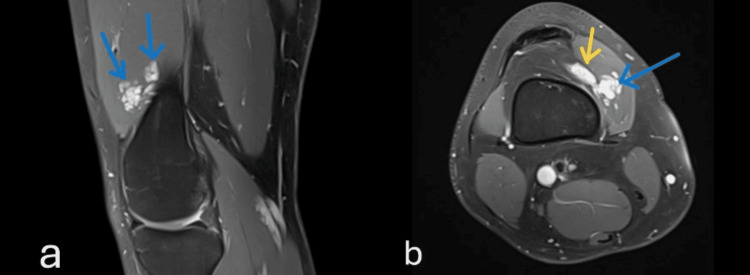
MRI of the right knee a) Proton Density Fat-Saturated sagittal sections imaging and b) Proton Density Fat-Saturated axial sections imaging showing well-defined, non-homogeneous, hypertension signals of the hemangioma (intramuscular mass with blue arrow and intrasynovial mass with yellow arrow)

An 18-year-old male presented to our practice with pain in his right knee. There was no history of trauma, infection, or any systemic disease. Physical examination revealed a painful palpable mass at the lower third of the vastus medialis. Knee flexion was restricted due to pain in the lower third of the thigh and around the knee joint. Preoperative MRI of the right knee showed a vascular malformation within the fibers of the vastus medialis muscle in connection with the knee joint, with dimensions 3.8 × 3.1 × 1.7 cm (Fig. 1).

**Figure 2:**
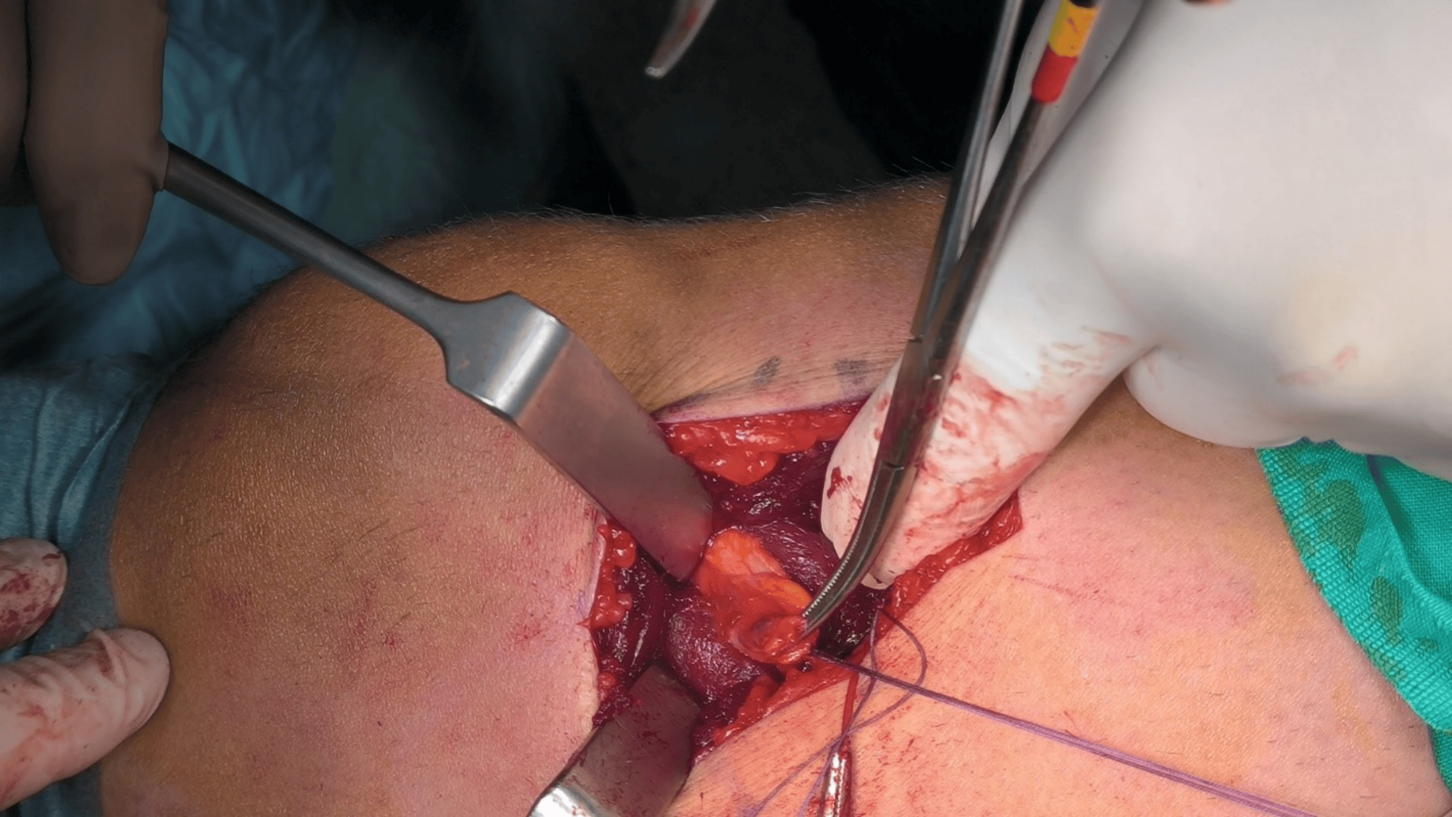
Intraoperative image showing hemangiomatous lesion in the vastus medialis muscle

We carefully performed open resection of the lesion. Intraoperatively, the mass was situated deep in the vastus medialis muscle in contact with the knee joint. The feeding vessel was the anastomosis of the musculoarticular branch of the descending genicular artery with the superior medial genicular artery. We performed careful ligation of the vessels and excision of the whole mass (Fig. 2).

**Figure 3:**
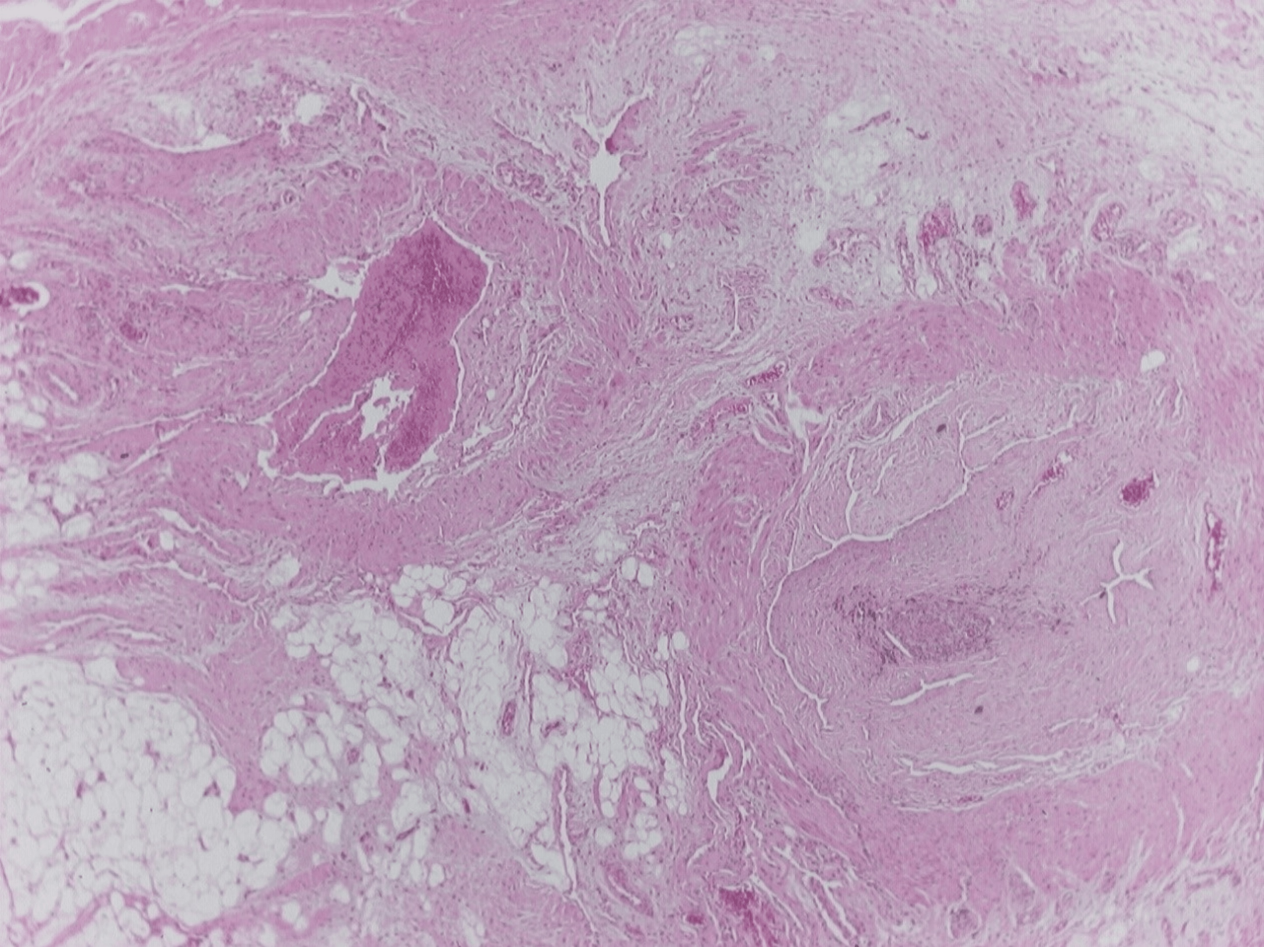
Histopathology of the excisional biopsy with fibrous tissue and papillary proliferation of blood vessels and organized thrombus

Histological examination revealed fibrovascular connective tissue with papillary formations lined by a single layer of endothelial cells. No features suggestive of malignancy were found. Histopathological findings confirmed the diagnosis of intravascular papillary endothelial hyperplasia - Masson hemangioma (Fig. 3).

**Figure 4:**
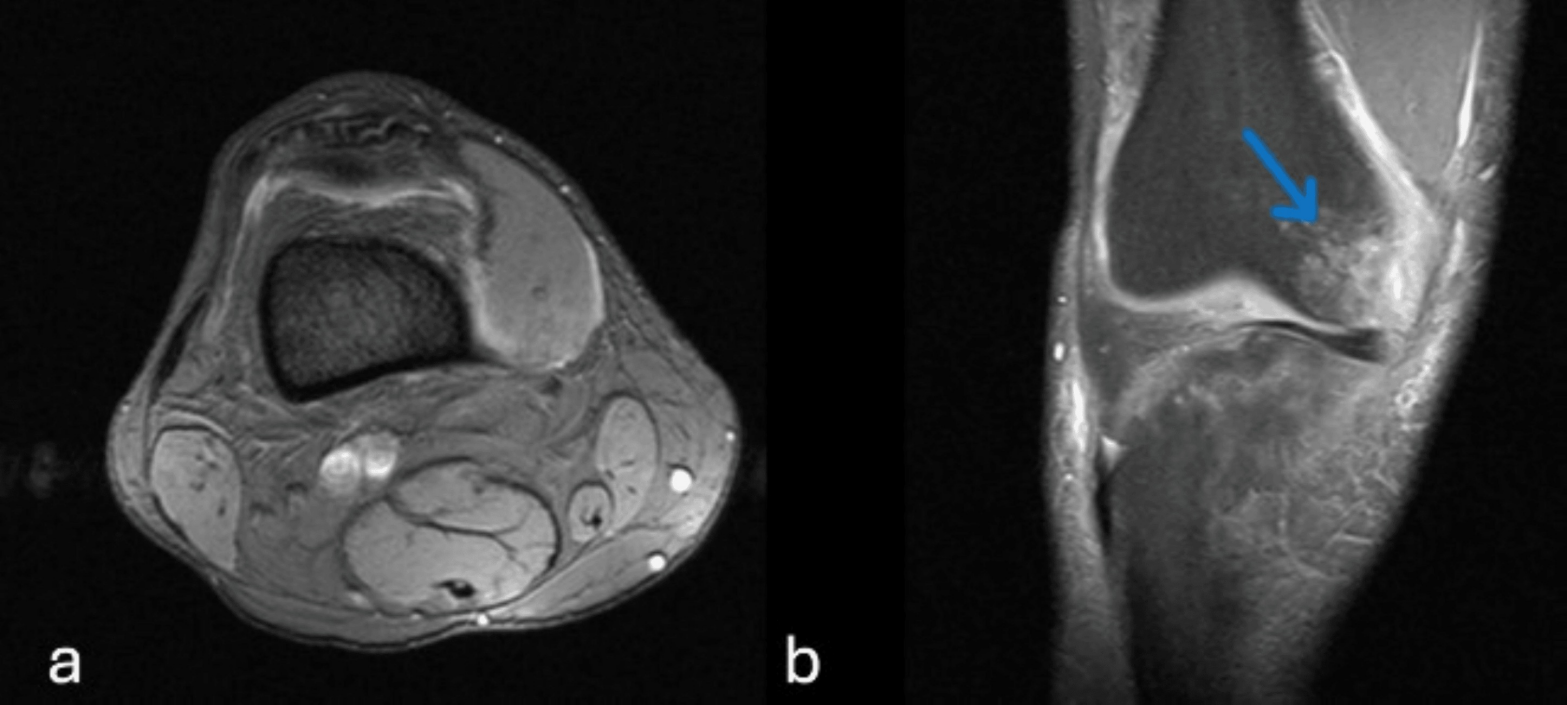
MRI of the right knee a) axial section shows no evidence of hemangioma in the vastus medialis muscle or intra-articularly; b) coronal section shows a bruise involving the medial femoral condyle (blue arrow points to the hyperintense signal)

One month later, the patient regained full range of motion in his knee and was able to return to his previous sports activities without pain or restriction. Ten months later, he experienced a knee sprain during sports activities. An MRI was performed to evaluate the nature of the injury, particularly to identify any alterations such as ligament or meniscal tears or cartilage damage. The MRI showed no evidence of hemangioma in the vastus medialis muscle or within the joint. The only finding was bone bruising of the medial femoral condyle (Fig. 4). There was a good functional outcome and no recurrence of the hemangioma.

## Discussion

In 1923, Pierre Masson was the first to describe what is now known as Masson’s hemangioma, originally identifying it as “hemangioendothelioma vegetant intravascular” [6]. He characterized it as a benign vascular lesion that can closely mimic malignant tumors such as angiosarcoma [7]. Over time, it has been referred to by various names, including “papillary fibroendothelioma,” “Masson’s pseudoangiosarcoma,” and “Masson’s lesion.” In 1976, Clearkin and Enzinger introduced the term intravascular papillary endothelial hyperplasia (IPEH), which has since become the most widely used designation in literature [8].

IPEH is a benign vascular lesion characterized by reactive endothelial cell proliferation within the lumen of blood vessels, often in response to vascular injury or thrombosis. Histologically, it presents as a dilated vessel containing papillary structures composed of plump, hyperplastic endothelial cells overlying fibrous tissue. These papillae are typically associated with organizing thrombi and fibrin deposition. The lesion lacks features of malignancy, such as necrosis, significant pleomorphism, high mitotic activity, or solid cellular areas, which helps distinguish it from angiosarcoma, a malignant vascular tumor marked by infiltrative growth, pronounced cellular atypia, frequent mitoses, and a poor prognosis. Immunohistochemical staining with markers such as CD31 and CD34 confirms the vascular origin of IPEH, while the proliferative index can further support an accurate diagnosis. Although IPEH is predominantly intravascular, extravascular hematoma organization may also be observed. Proper differentiation from angiosarcoma is crucial, as IPEH is curable through local excision, whereas angiosarcoma often requires more aggressive treatment due to its metastatic potential [4, 7].

IPEH most commonly arises in the skin and soft tissues, particularly in the head, neck, and extremities, where it typically involves small to medium-sized veins. It is often associated with preexisting vascular malformations or thrombi in these regions. Less frequently, IPEH has also been reported in the oral and orbital cavities. Extremely rare cases of involvement have been reported in a variety of organs and anatomical sites, including the brain, spine, heart, liver, kidney, bone, knee joint, urethra, and genitalia. These patterns of occurrence underscore the lesion’s preference for superficial vascular structures [7].

Case reports have documented occurrences of IPEH throughout the lower extremity in the following locations. It has been found in the right common femoral vein [9]. Two cases were reported on the thigh: one in the middle of the right thigh [10] and the other in the lower third of the left thigh [1]. Another case was found only in the synovium of the left knee joint [11]. One was located in the medial part of the proximal tibia [12]. Four cases occurred in the foot: one adherent to a branch of the saphenous nerve [13], one on the dorsal aspect of the right forefoot above the second metatarsal bone [7], one on the dorsal aspect of the right midfoot, specifically superficial to the extensor tendons of the toes and surrounding the long extensor of the hallux, near the dorsal artery of the foot [14], and one in the dorsal lateral part of the fifth metatarsal head of the right foot [15]. One additional case was reported on the medial aspect of the right fifth toe [16].

The descending genicular artery originates from the distal femoral artery approximately 12-17 cm above the knee joint line in the region of the adductor hiatus. It descends medially to the distal femoral diaphysis and the medial femoral condyle. Its size is often inversely related to the superior medial genicular artery. It has a highly variable branching pattern that usually splits into two or three major branches. The muscular and osteoarticular branches often share a common trunk and are also described collectively as the musculoarticular branch. It anastomoses with the superior medial genicular artery, superior lateral genicular artery, and inferior medial genicular artery [17].

## Conclusions

In conclusion, IPEH is a rare but benign vascular lesion that can closely mimic malignant tumors such as angiosarcoma, both clinically and histologically. Accurate diagnosis is crucial to avoid unnecessary aggressive treatment, as IPEH is curable with local excision. Its distinction from malignancies relies heavily on histopathological evaluation, supported by imaging and cytological studies. Awareness of its presentation and characteristics is essential for appropriate management and prognosis.
